# Anti-CD20 B-cell depletion enhances monocyte reactivity in neuroimmunological disorders

**DOI:** 10.1186/1742-2094-8-146

**Published:** 2011-10-26

**Authors:** Klaus Lehmann-Horn, Eva Schleich, Deetje Hertzenberg, Alexander Hapfelmeier, Tania Kümpfel, Nikolas von Bubnoff, Reinhard Hohlfeld, Achim Berthele, Bernhard Hemmer, Martin S Weber

**Affiliations:** 1 Department of Neurology, Technische Universität München, Munich, Germany; 2 Institute of Medical Statistics and Epidemiology, Technische Universität München, Munich, Germany; 3 Institute of Clinical Neuroimmunology, Ludwig-Maximilians-University, Munich, Germany; 4 Department of Internal Medicine, Technische Universität München, Munich, Germany

**Keywords:** multiple sclerosis, neuromyelitis optica, anti-CD20, B-cell regulation, monocytes, experimental autoimmune encephalomyelitis

## Abstract

**Background:**

Clinical trials evaluating anti-CD20-mediated B-cell depletion in multiple sclerosis (MS) and neuromyelitis optica (NMO) generated encouraging results. Our recent studies in the MS model experimental autoimmune encephalomyelitis (EAE) attributed clinical benefit to extinction of activated B-cells, but cautioned that depletion of naïve B-cells may be undesirable. We elucidated the regulatory role of un-activated B-cells in EAE and investigated whether anti-CD20 may collaterally diminish regulatory B-cell properties in treatment of neuroimmunological disorders.

**Methods:**

Myelin oligodendrocyte glycoprotein (MOG) peptide-immunized C57Bl/6 mice were depleted of B-cells. Functional consequences for regulatory T-cells (Treg) and cytokine production of CD11b^+ ^antigen presenting cells (APC) were assessed. Peripheral blood mononuclear cells from 22 patients receiving anti-CD20 and 23 untreated neuroimmunological patients were evaluated for frequencies of B-cells, T-cells and monocytes; monocytic reactivity was determined by TNF-production and expression of *signalling lymphocytic activation molecule *(SLAM).

**Results:**

We observed that EAE-exacerbation upon depletion of un-activated B-cells closely correlated with an enhanced production of pro-inflammatory TNF by CD11b^+ ^APC. Paralleling this pre-clinical finding, anti-CD20 treatment of human neuroimmunological disorders increased the relative frequency of monocytes and accentuated pro-inflammatory monocyte function; when reactivated ex vivo, a higher frequency of monocytes from B-cell depleted patients produced TNF and expressed the activation marker SLAM.

**Conclusions:**

These data suggest that in neuroimmunological disorders, pro-inflammatory APC activity is controlled by a subset of B-cells which is eliminated concomitantly upon anti-CD20 treatment. While this observation does not conflict with the general concept of B-cell depletion in human autoimmunity, it implies that its safety and effectiveness may further advance by selectively targeting pathogenic B-cell function.

## Background

Accumulating evidence suggests that in the pathogenesis of multiple sclerosis (MS) and neuromyelitis optica (NMO), B-cells, plasma cells and self-reactive antibodies play an essential pathogenic role. In MS, an oligoclonal antibody response generated by a limited repertoire of activated B-cells remains a hallmark diagnostic finding in the cerebrospinal fluid (CSF)[1]. While target and pathogenic relevance of this humoral response is still under debate [2], autoantibodies against aquaporin-4 (AQP-4) allow to distinguish NMO from other central nervous system (CNS) demyelinating conditions, promote development of NMO-like lesions in animal models [3] and may correlate with progression of NMO itself [4]. Besides developing into plasma cells secreting self-reactive antibodies, antigen-activated B-cells may directly contribute to development of neuroimmunological disease by transporting, processing and presenting antigen to self-reactive T-cells. As activated T-cells in return promote differentiation of B-cells and isotype switching of plasma cells, the interaction of auto-reactive B- and T-cells may foster each other's development in progression of CNS autoimmune disease.

Based on these pathogenic B-cell properties, substantial interest has developed for testing anti-CD20 antibodies (rituximab, ocrelizumab, ofatumumab) in MS and NMO. These antibodies deplete immature and mature B-cells, but spare CD20-negative plasma cells. The retrospective analysis of 25 NMO patients receiving rituximab demonstrated a reduction in attack frequency with subsequent clinical stabilization [5]. While one study suggested that clinical benefit may relate to a decline in anti-AQP-4 antibody titers [4], it is unclear whether depletion of CD20^+ ^AQP4-specific plasma cell precursors provides the sole and entire basis for therapeutic benefit of anti-CD20 in NMO [6]. Clinical trials testing anti-CD20 rituximab in MS generated encouraging results as well. In relapsing-remitting MS, treatment with rituximab or its humanized successor ocrelizumab led to a rapid decline in newly developing inflammatory CNS lesions [7,8]; in treatment of primary progressive MS, rituximab reduced lesion formation in a subgroup of younger patients with active CNS inflammation [9]. Immunological analyses revealed that anti-CD20 B-cell depletion diminished proliferation and pro-inflammatory differentiation of peripheral T-cells [10]; further, rituximab-treatment was associated with a reduced number of B-cells, but also of T-cells within the CSF of patients with relapsing-remitting (RR)-MS [11]. Together, these findings highlight abrogation of B-cell-mediated T-cell activation as an important mechanism for the prompt effect of anti-CD20 treatment in CNS demyelinating disorders.

Notwithstanding these encouraging results, not all CD20^+ ^B-cells may actively contribute to progression of autoimmune disease. Animal models of human autoimmunity suggest that through provision of anti-inflammatory IL-10, naïve B-cells in contrast regulate autoimmune responses [12] and control pro-inflammatory differentiation of other antigen presenting cells (APC) [13]. Accumulating evidence suggests that equivalent regulatory B-cell properties exist in humans [14]. In a recent report, Iwata and colleagues described a subset of regulatory IL-10 producing B-cells in various autoimmune conditions, including MS with an overall frequency and IL-10 production comparable to healthy individuals [15]. Functionally, these regulatory B-cells inhibited TNF-release of monocytes isolated from the identical patient, further fueling the concept that regulatory B-cell subsets control pro-inflammatory activity of other APC populations.

Our recent study testing anti-CD20 treatment in an animal model of MS, revealed that B-cell depletion exacerbated experimental autoimmune encephalomyelitis (EAE) induced by the short T-cell determinant myelin-oligodendrocyte glycoprotein (MOG) peptide (p)35-55, a setting in which B-cells are not required or involved in a pathogenic manner [16]. One aim of our current investigation was thus to elucidate the immunological mechanisms for deterioration of EAE in this setting. We demonstrate that EAE-exacerbation upon depletion of un-activated B-cells closely correlates with an enhanced production of pro-inflammatory TNF by CD11b^+ ^APC. In light of these preclinical findings and the newly established role of B-cell subsets in regulation of human autoimmunity, we further investigated whether anti-CD20 treatment may collaterally abolish B-cell regulatory properties in human neuroimmunological disorders. Paralleling our findings in EAE, we report that anti-CD20 treatment of MS and NMO is associated with an accentuation of pro-inflammatory monocyte function, providing the first evidence that besides abrogation of pathogenic B-cell function, anti-CD20 diminishes B-cell regulation of myeloid APC.

## Methods

### Subjects and specimens

This study was approved by the local ethics committee of the Technische Universität München. After informed consent, subjects were enrolled in four groups: rituximab-treated patients with neuroimmunological disorders, untreated patients with neuroimmunological disorders, rituximab-treated B-cell lymphoma patients and untreated patients with other non-inflammatory disorders (table 1 and additional file 1). Patients had not received corticosteroids within 3 months or any immunosuppressive, immunomodulatory or chemo- therapy within 6 months prior to enrollment.

**Table 1:** Characteristics of patients with neuroimmunological disorders and analysis of peripheral blood mononuclear cells.

Neuroimmunological patients		α-CD20	control	p-values
**number of subjects**		22	23	

**gender**	**female**	18	18	
	**male**	4	5	

**age [years]**	**mean (min. - max.)**	45 (18-69)	42 (17-69)	

**α-CD20 treatment duration [months] **	**mean (min. - max.)**	15 (2-47)	n.a.	

**disorder**	**MS/CIS**	8	22	
	**NMO**	11	1	
	**Myasthenia gravis**	2	0	
	**autoimmune neuropathy**	1	0	

**CD19^+ ^of all PBMCs**	**[mean % +/- SEM]**	0.2 (+/-0.1)	7.9 (+/-1.1)	<0.0001
**CD4^+ ^of all PBMCs**	"	37.7 (+/-2.5)	35.9 (+/-1.5)	0.420
**CD8^+ ^of all PBMCs**	"	16.8 (+/-1.8)	15.5 (+/-1.1)	0.768
**CD14^+ ^of all PBMCs**	"	22.3 (+/-2.5)	16.4 (+/-1.4)	0.159
**CD4^+ ^of all CD4^+^/CD8^+^**	"	69.2 (+/-2.2)	69.8 (+/-1.9)	0.803
**CD4^+ ^of all CD4^+^/CD8^+^/CD14^+^**	"	48.6 (+/-2.9)	53.2 (+/-2.0)	0.271
**CD8^+ ^of all CD4^+^/CD8^+^/CD14^+^**	"	21.3 (+/-2.0)	22.9 (+/-1.5)	0.370
**CD14^+ ^of all CD4^+^/CD8^+^/CD14^+^**	"	30.0 (+/-3.7)	23.9 (+/-2.1)	0.163
**CD25^+ ^CD127^- ^of all CD4^+^**	**[median % with 20/80% percentile]**	6.8 (5.5-8.4)	5.2 (4.3-6.8)	0.022

Anti-CD20-treated and untreated (control) patients were age- and sex-matched. Frequencies of leucocyte subpopulations are indicated as percentage of all peripheral blood mononuclear cells (PBMCs), and as percentage of CD4^+^, CD4^+^/CD8^+ ^or CD14^+^CD4^+^/CD8^+ ^PBMCs to "normalize" for treatment-related absence of B-cells. MS = multiple sclerosis; CIS = clinically isolated syndrome; NMO = neuromyelitis optica.

### FACS staining of leucocyte subpopulations and monocytic activation

PBMCs were stained for CD19, CD4, CD14, CD25, CD127, SLAM/CD150 (all BD Bioscience) or CD8a (eBioscience). FACS staining was analyzed on a Cyan ADP9C using software Summit 4.3 (Beckmann Coulter). PBMCs were stimulated with lipopolysaccharid (LPS) and SLAM-expression of CD14^+ ^monocytes was evaluated 24 hours thereafter. Frequency of CD14^+ ^monocytes expressing SLAM was determined as shown in additional file 2.

### Analysis of TNF-producing monocytes

Magnetically activated cell sorting (MACS)-separated monocytes (positive selection using CD14 antibodies, Miltenyi Biotec; purity >90%) were plated in TNF capture antibody-precoated Multi-Screen Filter Plates (Millipore) in triplicates (3,000 cells/well) and stimulated with LPS for 18 hours. Plates were washed and incubated successively with TNF detection antibody, streptavidin-alkaline phosphatase and BCIP/NBT substrate. Plates were analyzed with an automated imaging system and software (AID EliSpot reader and software, Autoimmun Diagnostika).

### Mice, EAE induction and depletion of B-cells and regulatory T-cells

All murine experiments were carried out as approved by the government of Upper Bavaria (protocol number 55.2-1-54-2531-67-09). C57BL/6 female mice were immunized with 100 μg MOG p35-55 (Auspep, Australia) in Complete Freund's Adjuvant (CFA) followed by 200 ng of pertussis toxin (PTX) i.p. at the day of immunization and 2 days thereafter. Mice were assessed for signs of EAE as described previously [16]. Mice received weekly i.p. injections of 200μg of murine anti-CD20 or isotype-control starting 21 days prior to immunization (provided by Genentech, South San Francisco, USA) and 500μg of anti-mouse CD25 antibody (BioXcell, West Lebanon, USA) or isotype control 5 and 3 days prior to EAE induction. In unimmunized mice, anti-CD25 antibodies are commonly used to deplete regulatory T cells as they represent the majority of CD25^+ ^cells in naïve mice. Results are representative of 3 separate experiments.

### Detection of TNF produced by murine monocytes

12 days after immunization, MACS-purified splenic monocytes (positive selection using CD11b antibodies, Miltenyi Biotec; purity >90%) were stimulated with the indicated concentrations of LPS. After 24 hours, supernatants were collected and analyzed for murine TNF by ELISA (R&D Systems). Plates were read at 450 nm wavelength by a Tecan Genios plate reader and analyzed using Magellan6 software.

### Statistical analysis

As frequency of regulatory T-cells followed a skewed distribution, the Mann-Whitney U-Test was used for comparisons. Frequency of monocytes was distributed normally and analyzed by t-Test. Variability of monocytic SLAM expression was compared using the Siegel-Tukey test, capable to deal with non-normal data. Variability of TNF-producing monocytes in anti-CD20 treated vs. untreated patients was compared using the F-Test based on a normal distribution of values. All statistical tests were two-sided and conducted in an explorative manner on a 5% level of significance. Descriptive statistics for continuous, normally distributed data are given by the mean, its standard error (SEM) or the range (min. - max.). Skewed data is presented by the median as well as 20% and 80% percentiles. Categorical data is summarized by absolute and relative frequencies.

## Results and Discussion

**Figure 1:**
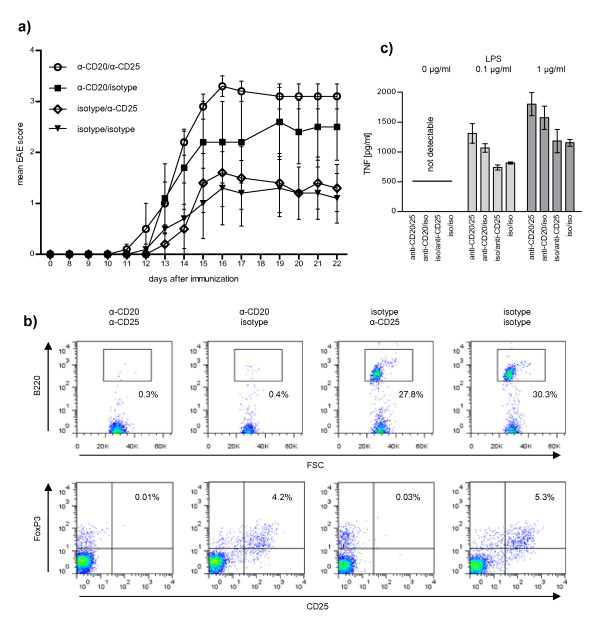
**Regulatory B-cells and regulatory T-cells control EAE independent of each other - B-cell depletion-associated EAE exacerbation correlates with enhanced TNF secretion of CD11b^+ ^cells**. C57Bl/6 mice were injected with 200 μg anti-CD20 and/or 500 μg anti-CD25 and/or the respective isotype control in the combinations indicated prior to immunization with MOG p35-55. **a) **Shown are mean group scores of EAE severity ± SEM (5 mice/group). **b) **Depletion of B-cells and/or regulatory T-cells was evaluated by FACS staining for B220 (upper panel) or CD25/FoxP3 (lower panel, gated on CD4^+^). Shown are FACS stainings of inguinal lymph node cells obtained from representative mice prior to immunization. **c) **Secretion of TNF by splenic CD11b^+ ^monocytes upon stimulation with LPS was evaluated by ELISA (mean of triplicates ± within subject standard deviation; 2 mice/group).

In our previous study, anti-CD20-mediated depletion of un-activated B-cells exacerbated MOG p35-55-induced EAE which was associated with a reduced frequency of regulatory T-cells (Treg) and a pronounced pro-inflammatory differentiation of myeloid CD11b^+ ^APC. In order to dissect the relative responsibility of either effect for clinical deterioration, we utilized an anti-CD25 Treg-depleting antibody to neutralize for alterations in Treg frequency. Prior to disease induction, mice were injected with anti-CD25, anti-CD20 or a combination of both antibodies. As expected, anti-CD20-mediated B-cell depletion exacerbated disease severity (Figure 1a+b). Depletion of Treg alone only modestly worsened disease, whereas Treg-depleted mice substantially deteriorated when B-cells were depleted in addition to Treg. These findings indicate that clinical exacerbation of MOG peptide-induced EAE upon B-cell depletion is not explained by a treatment-related reduction in Treg frequency and confirm that regulatory B- and T-cells control CNS autoimmune disease independent of each other [17].

We investigated next whether alternatively, elimination of B-cell-mediated regulation of APC activity may account for anti-CD20-associated worsening of peptide-induced EAE. CD11b^+ ^APC were isolated from all four groups of mice and evaluated for production of the pro-inflammatory hallmark cytokine TNF. As indicated in Figure 1c, in all mice depleted of B-cells, remaining CD11b^+ ^cells produced increased levels of pro-inflammatory TNF. This effect was further accelerated when mice were in addition depleted of Treg, resulting in a close correlation between the relative increase in monocytic TNF release and the extent of clinical deterioration. In our previous study, elevated TNF production by CD11b^+ ^cells resulted in an enhanced ability of these APC to generate encephalitogenic Th1 and Th17 cells [16]. TNF was further shown to direct migration of these cells within the CNS, facilitating early initiation of CNS autoimmune disease [18]. Collectively, these findings support the conclusion that in EAE, naive B-cells regulate CD11b^+ ^APC and highlight an enhanced pro-inflammatory APC function as explanation for exacerbation of CNS autoimmune disease upon depletion of naïve B-cells.

**Figure 2:**
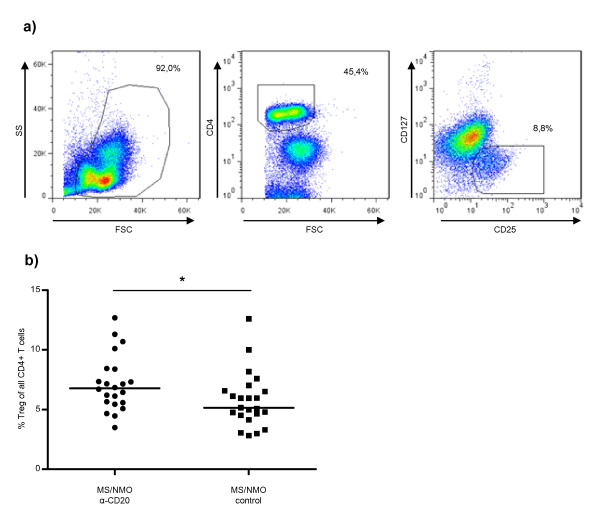
**In treatment of human neuroimmunological disorders, anti-CD20-mediated B-cell depletion is associated with an increase in the frequency of regulatory T-cells**. Peripheral blood mononuclear cells (PBMCs) were isolated from anti-CD20-treated or untreated patients with neuroimmunological disorders (see table 1). Frequency of regulatory T-cells is indicated as percentage of CD4^+^CD25^+^CD127^- ^within all CD4^+ ^T-cells (**a+b**, black lines represent median; * p = 0.022).

Based on these pre-clinical findings, we investigated the immunological consequences of anti-CD20 treatment in human neuroimmunological disorders. Peripheral blood mononuclear cells (PBMCs) were isolated from 22 rituximab-treated patients with MS, NMO, myasthenia gravis or autoimmune neuropathy and compared to PBMCs from 23 age- and sex-matched untreated patients (see table 1). All rituximab-treated subjects showed a virtually complete depletion of peripheral CD19^+ ^B-cells whereas PBMCs from control patients contained a mean frequency of 7.9 ± 1.1% B-cells (table 1 ). All other leucocyte subpopulations were compared as percentages of CD4^+^, CD4^+^/CD8^+ ^or CD14^+^/CD4^+^/CD8^+ ^PBMCs in order to "normalize" for treatment-related absence of B-cells. While the overall frequency of CD4^+ ^and CD8^+ ^cells remained unchanged, anti-CD20 treatment raised the relative frequency of CD4^+^CD25^+^CD127^- ^Treg within all CD4^+ ^T-cells cells (6.8, 5.5-8.4 20/80% percentile, vs. 5.2, 4.3-6.8 20/80% percentile; table 1+Figure 2; p = 0.022). This novel finding needs to be supported by future evaluation of absolute numbers and functional capacity of Treg upon anti-CD20 treatment; nonetheless, several clinical trials in other autoimmune diseases also provided evidence that anti-CD20 may augment frequency and/or function of Treg [18-21]. Together, these observations could indicate that restitution of a disease-intrinsically impaired regulatory T-cell function may be an additional mechanism by which anti-CD20 mediates broad clinical benefit in human autoimmune disease.

**Figure 3:**
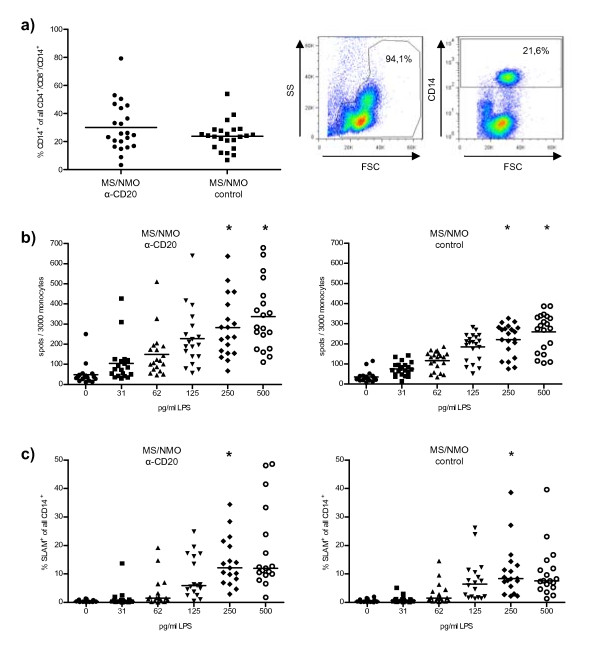
**In treatment of human neuroimmunological disorders, anti-CD20 treatment is associated with an enhanced pro-inflammatory activity of monocytes**. Peripheral blood mononuclear cells (PBMCs) were isolated from anti-CD20-treated or untreated patients with neuroimmunological disorders (see table 1) **a) **Frequency of monocytes was determined as percentage of CD14^+ ^cells within all CD4^+^/CD8^+^/CD14^+ ^PBMCs. Black lines represent mean; p = 0.163. **b) **MACS-purified monocytes were stimulated with the indicated concentrations of LPS; TNF secretion was evaluated by ELISPOT (indicated as frequency of TNF-producing cells/3,000 monocytes; black lines represent mean; *p < 0.05) **c) **PBMCs were stimulated with the indicated concentrations of LPS; monocytic expression of *signalling lymphocytic activation molecule *(SLAM) was evaluated by FACS (indicated as percentage of SLAM^+ ^cells within all CD14^+ ^monocytes; black lines represent median; * p = 0.034).

The main purpose of this translational approach was to investigate whether anti-CD20 treatment of human neuroimmunological disorders may concomitantly abrogate B-cell regulation of other APC. As shown in table 1 and Figure 3a, PBMCs from B-cell-depleted patients showed a trend towards an increase in the frequency of CD14^+ ^monocytes (30.0 ± 3.7% vs. 23.9% ± 2.1%; p = 0.163). In order to compare pro-inflammatory monocyte reactivity, we evaluated LPS-induced release of TNF and expression of *signaling lymphocytic activation molecule *(SLAM), an activation marker which physiologically serves as a co-stimulatory molecule promoting development of pro-inflammatory T-cells [22]. As shown in Figure 3b, a higher frequency of monocytes from B-cell-depleted patients released TNF (e.g. 281.5 ± 34.8 vs. 222.0 ± 17.2 per 3000 monocytes at 250 pg/ml LPS). Compared to control patients, samples from B-cell-depleted patients were distributed over a wide range of values, which is reflected by a significantly greater variability of monocytic TNF production (p < 0.05 at 250 and 500 pg/ml LPS). Correspondingly, the group of anti-CD20-treated patients contained a higher number of samples in which monocytes expressed activation-induced SLAM at a high frequency, again resulting in a greater variability of monocytic SLAM expression in B-cell-depleted patients (Figure 3c; p = 0.034 at 250 pg/ml LPS). Ongoing studies aim to elaborate whether individual patients longitudinally experience an increase in monocytic activation and/or frequency of Treg upon therapeutic B cell depletion. Importantly, within the group of anti-CD20-treated neuroimmunological patients monocytic expression of TNF and SLAM did not correlate with the underlying disorder (e.g. MS vs. NMO), age or treatment duration (data not shown). In contrast, unleashing of pro-inflammatory APC activity upon depletion of B-cells appeared to relate to the stimulating milieu of underlying chronic inflammation: compared to age- and sex-matched non-inflammatory controls, PBMCs from anti-CD20-treated B-cell lymphoma patients contained a higher frequency of Treg (additional file 3 ), but showed no enhanced monocytic TNF release or SLAM expression (additional file 3). Taken together, these findings indicate that control of APC activity is a counterbalancing B-cell property in immunological disorders, which is eliminated by anti-CD20 treatment.

While the majority of patients with neuroimmunological disorders clearly benefit from anti-CD20 treatment [5,7,8], few cases have been reported in which autoimmune disease progression appeared to be promoted. In a patient with anti-MAG polyneuropathy disability worsened within weeks following anti-CD20 treatment [23]; in a small study with individuals with anti-MAG polyneuropathy, 8 patients clinically stabilized or improved while one patient markedly deteriorated upon B-cell depletion [24]. In another report, a patient with myasthenia gravis developed ulcerative colitis while on anti-CD20 treatment [25]. A patient with anti-MAG polyneuropathy and secondary-progressive MS showed an improvement of polyneuropathy symptoms, but experienced 2 persistently disabling MS relapses [26]; another patient with NMO severely progressed while on anti-CD20 therapy [27]. In light of our new findings, and having in mind that monocytic TNF and SLAM expression strongly varied among anti-CD20-treated patients with only few individuals displaying substantially elevated levels, it will be crucial to investigate whether such assumed occasional promotion of autoimmunity may correlate with an enhanced pro-inflammatory APC activity upon anti-CD20 treatment.

## Conclusions

In conclusion, we herein provide novel evidence that besides abrogation of pathogenic B-cell function, anti-CD20 treatment eliminates preexisting B-cell regulation in human autoimmunity. In treatment of NMO and MS, this observation in conjunction with our EAE findings could indicate that individual patients with minor counter-balancing pathogenic B-cell involvement may not benefit or even deteriorate upon pan-B-cell depletion via CD20. Whereas our study does not conflict with the projected general potential of B-cell depletion in treatment of autoimmune disorders, it cautions that its indication should be assessed individually and supports further development of this therapeutic approach to selectively target pathogenic B-cell function.

## List of abbreviations

APC: antigen presenting cell; AQP-4: aquaporin-4; CFA: Complete Freund's Adjuvant; CIS: clinically isolated syndrome; CNS: central nervous system; CSF: cerebrospinal fluid; EAE: experimental autoimmune encephalomyelitis; ELISA: enzyme linked immunosorbent assay; ELISPOT: enzyme linked immuno spot technique; FACS: fluorescence activated cell sorting; IL-10: interleukin 10; LPS: lipopolysaccharid; MACS: magnetically activated cell sorting; MAG: myelin associated glycoproteins; MOG: myelin oligodendrocyte glycoprotein; MS: multiple sclerosis; NMO: neuromyelitis optica; PBMC: Peripheral blood mononuclear cell; RR-MS: relapsing-remitting multiple sclerosis; SEM: standard error of the mean; SLAM: signalling lymphocytic activation molecule; TNF: tumor necrosis factor.

## Competing interests

The authors declare that they have no competing interests.

## Authors' contributions

KL-H performed experiments, interpreted data and contributed in drafting the manuscript, ES performed experiments and interpreted data; DH, AH and TK interpreted the data; NvB contributed to conception and design of the study; RH and AB have been involved in drafting the manuscript; BH revised the manuscript critically for important intellectual content; MSW performed experiments, designed the research, interpreted the data and wrote the manuscript. All authors have given final approval of the version to be published.

## Supplementary Material

Additional file 1 **Characteristics of patients with B-cell lymphoma or various non-inflammatory neurological disorders and analysis of peripheral blood mononuclear cells**. Anti-CD20 treated B-cell lymphoma and untreated non-inflammatory (control) patients were age- and sex-matched. Frequencies of leucocyte subpopulations are indicated as percentage of all peripheral blood mononuclear cells (PBMCs) and as percentage of CD4^+^, CD4^+^/CD8^+ ^or CD14^+^CD4^+^/CD8^+ ^PBMCs to "normalize" for treatment-related absence of B-cells. Click here for file

Additional file 2 **Activation-induced monocytic expression of *signalling lymphocytic activation molecule *(SLAM)**. PBMCs were stimulated with increasing concentrations of LPS. Expression of SLAM was evaluated by FACS (gated on CD14^+ ^monocytes); non-stimulated PBMCs served as base value and gates were set accordingly. Click here for file

Additional file 3 **In treatment of B-cell lymphoma, anti-CD20-mediated B-cell depletion is associated with an increased frequency of regulatory T-cells but not with an enhanced pro-inflammatory activity of monocytes**. Peripheral blood mononuclear cells (PBMCs) were isolated from anti-CD20-treated patients with B-cell lymphoma or untreated control patients with non-inflammatory neurological disorders (see additional file 1). **a) **The frequency of regulatory T-cells is indicated as percentage of CD4^+^CD25^+^CD127^- ^within all CD4^+ ^T-cells (black lines represent the median within each group; * = p < 0.001). **b) **The frequency of monocytes is indicated as the percentage of CD14^+ ^cells within the pool of PBMCs expressing CD4^+^/CD8^+^/CD14^+ ^(black lines represent the mean of each group; p = 0.194). **c) **MACS-separated monocytes were stimulated with the indicated concentrations of LPS; secretion of TNF was evaluated by ELISPOT. Shown is the number of TNF-producing cells/3,000 monocytes (black lines represent the mean of each group). **d) **PBMCs were stimulated with the indicated concentrations of LPS and monocytic expression of *signalling lymphocytic activation molecule *(SLAM) was evaluated by FACS. Indicated is the percentage of SLAM^+ ^cells within all CD14^+ ^monocytes (black lines represent the median of each group). Click here for file
