# Supplementary material for: Anti-CD20 B-cell depletion enhances monocyte reactivity in neuroimmunological disorders
Source: J Neuroinflammation. 2011 Oct 26;8:146. doi: 10.1186/1742-2094-8-146 (PMC3214191; doi:10.1186/1742-2094-8-146)
Supplement: Additional file 1 — Characteristics of patients with B-cell lymphoma or various non-inflammatory neurological disorders and analysis of peripheral blood mononuclear cells. Anti-CD20 treated B-cell lymphoma and untreated non-inflammatory (control) patients were age- and sex-matched. Frequencies of leucocyte subpopulations are indicated as percentage of all peripheral blood mononuclear cells (PBMCs) and as percentage of CD4+, CD4+/CD8+ or CD14+CD4+/CD8+ PBMCs to "normalize" for treatment-related absence of B-cells. [file 1742-2094-8-146-S1.PDF]

Nonimmunological patients		B-cell lymphoma α-CD20	control	p-values
number of subjects		23	23	
gender	female	10	10	
	male	13	13	
age [years]	mean (min. - max.)	63 (28-87)	61 (27-86)	
α-CD20 treatment duration [months]	mean (min. - max.)	15 (1-66)	n.a.	
disorder	B cell lymphoma	23	2	
	other	0	21	
$CD19^+$ of all PBMCs	[mean % +/- SEM]	0.1 (+/-0)	12.5 (+/-4.2)	<0.0001
$CD4^+$ of all PBMCs	”	21.3 (+/-2.5)	28.2 (+/-2.2)	0.037
$CD8^+$ of all PBMCs	”	21.8 (+/-2.1)	13.4 (+/-1.4)	0.006
$CD14^+$ of all PBMCs	”	28.6 (+/-2.4)	21.6 (+/-1.9)	0.048
$CD4^+$ of all $CD4^+/CD8^+$	”	48.1 (+/-3.9)	66.9 (+/-2.6)	0.001
$CD4^+$ of all $CD4^+/CD8^+/CD14^+$	”	29.2 (+/-3.2)	43.9 (+/-2.5)	0.002
$CD8^+$ of all $CD4^+/CD8^+/CD14^+$	”	30.5 (+/-2.9)	21.3 (+/-1.7)	0.031
$CD14^+$ of all $CD4^+/CD8^+/CD14^+$	”	40.3 (+/-3.4)	34.9 (+/-2.3)	0.194
$CD25^+$ $CD127^-$ of all $CD4^+$	[median % with 20/80% percentile]	9.0 (6.7-14.5)	6.0 (4.4-7.4)	<0.001
