# Supplementary figures and images for: Anti-CD20 B-cell depletion enhances monocyte reactivity in neuroimmunological disorders
Source: J Neuroinflammation. 2011 Oct 26;8:146. doi: 10.1186/1742-2094-8-146 (PMC3214191; doi:10.1186/1742-2094-8-146)

SLAM/CD150

non-stimulated

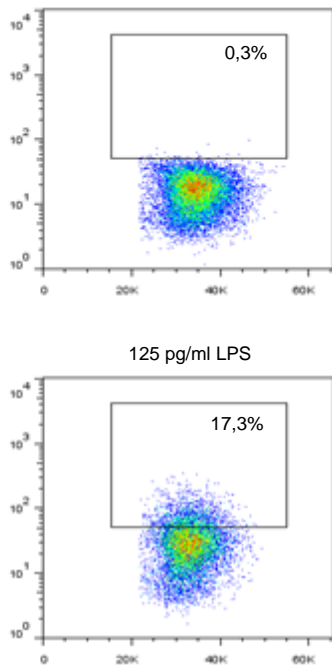

31 pg/ml LPS

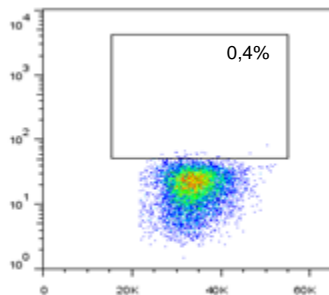

62 pg/ml LPS

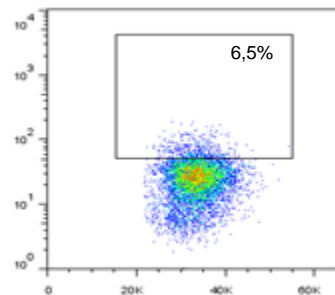

125 pg/ml LPS

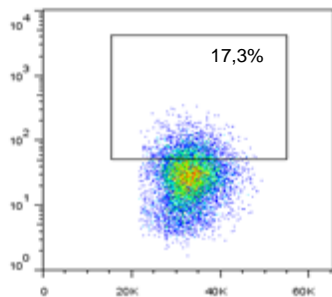

250 pg/ml LPS

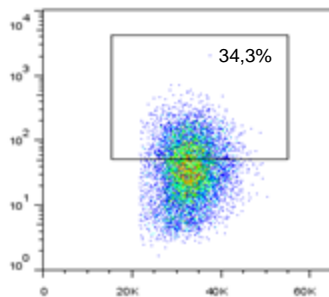

500 pg/ml LPS

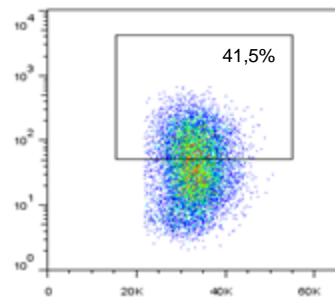

FSC

Supplement: Additional file 2 — Activation-induced monocytic expression of signalling lymphocytic activation molecule (SLAM). PBMCs were stimulated with increasing concentrations of LPS. Expression of SLAM was evaluated by FACS (gated on CD14+ monocytes); non-stimulated PBMCs served as base value and gates were set accordingly. [file 1742-2094-8-146-S2.PDF]

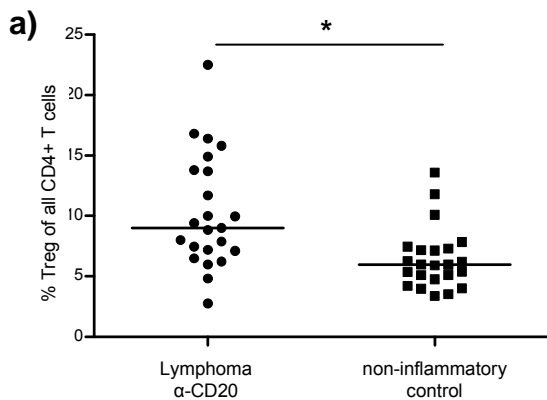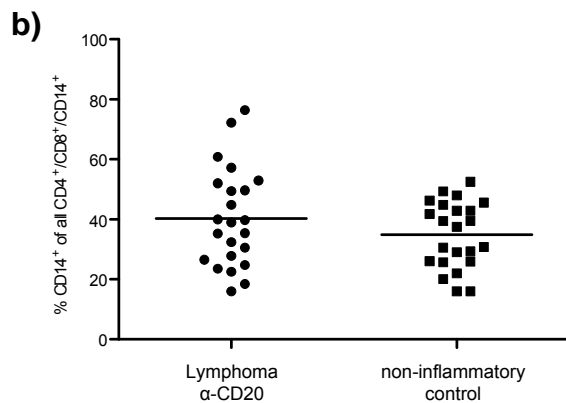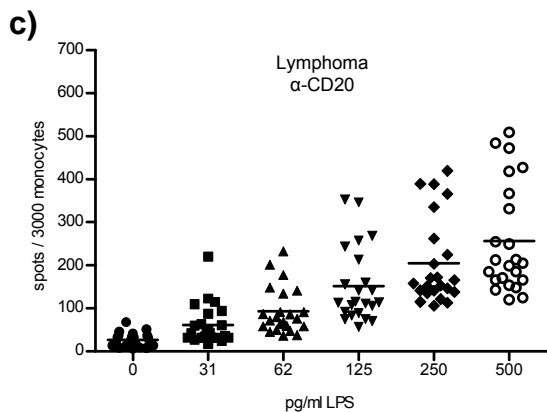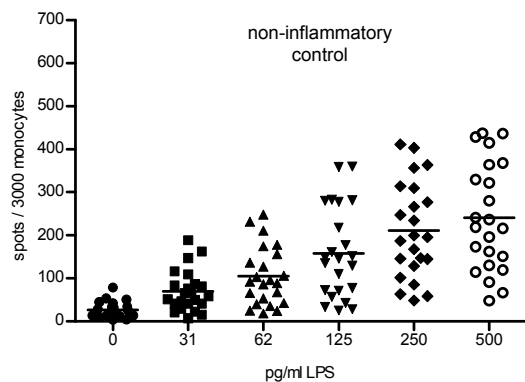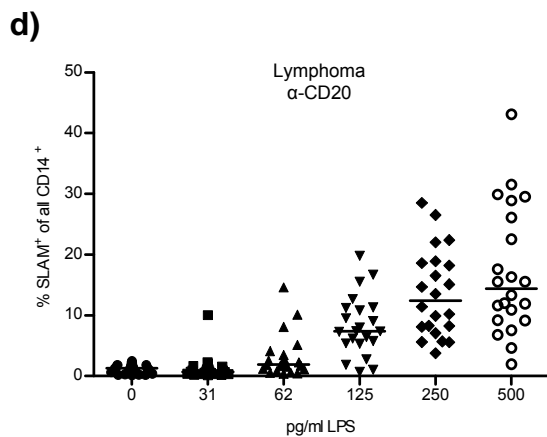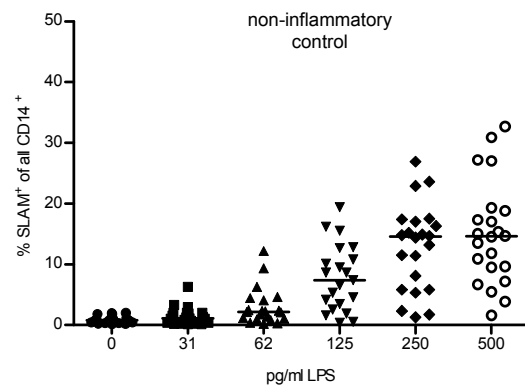

Supplement: Additional file 3 — In treatment of B-cell lymphoma, anti-CD20-mediated B-cell depletion is associated with an increased frequency of regulatory T-cells but not with an enhanced pro-inflammatory activity of monocytes. Peripheral blood mononuclear cells (PBMCs) were isolated from anti-CD20-treated patients with B-cell lymphoma or untreated control patients with non-inflammatory neurological disorders (see additional file 1). a) The frequency of regulatory T-cells is indicated as percentage of CD4+CD25+CD127- within all CD4+ T-cells (black lines represent the median within each group; * = p < 0.001). b) The frequency of monocytes is indicated as the percentage of CD14+ cells within the pool of PBMCs expressing CD4+/CD8+/CD14+ (black lines represent the mean of each group; p = 0.194). c) MACS-separated monocytes were stimulated with the indicated concentrations of LPS; secretion of TNF was evaluated by ELISPOT. Shown is the number of TNF-producing cells/3,000 monocytes (black lines represent the mean of each group). d) PBMCs were stimulated with the indicated concentrations of LPS and monocytic expression of signalling lymphocytic activation molecule (SLAM) was evaluated by FACS. Indicated is the percentage of SLAM+ cells within all CD14+ monocytes (black lines represent the median of each group). [file 1742-2094-8-146-S3.PDF]
